# The association between ibuprofen administration in children and the risk of developing or exacerbating asthma: a systematic review and meta-analysis

**DOI:** 10.1186/s12890-024-03179-3

**Published:** 2024-08-26

**Authors:** Luke Baxter, Maria M. Cobo, Aomesh Bhatt, Rebeccah Slater, Olutoba Sanni, Nutan Shinde

**Affiliations:** 1 Department of Paediatrics, University of Oxford, Oxford, UK; 2 Colegio de Ciencias Biologicas y Ambientales, Universidad San Francisco de Quito USFQ, Quito, Ecuador; 3 Reckitt, Dansom Lane, Hull, HU8 7DS UK; 4 Reckitt (Global Headquarters), Turner House, 103-105 Bath Road, Slough, Berkshire, SL1 3UH UK

**Keywords:** Ibuprofen, Children, Infants, Asthma, Wheezing, Cough, Hypersensitivity, Bronchospasm, Bronchoconstriction, Dyspnoea

## Abstract

**Background:**

Ibuprofen is one of the most commonly used analgesic and antipyretic drugs in children. However, its potential causal role in childhood asthma pathogenesis remains uncertain. In this systematic review, we assessed the association between ibuprofen administration in children and the risk of developing or exacerbating asthma.

**Methods:**

We searched MEDLINE, Embase, Cochrane Library, CINAHL, Web of Science, and Scopus from inception to May 2022, with no language limits; searched relevant reviews; and performed citation searching. We included studies of any design that were primary empirical peer-reviewed publications, where ibuprofen use in children 0–18 years was reported. Screening was performed in duplicate by blinded review. In total, 24 studies met our criteria. Data were extracted according to PRISMA guidelines, and the risk of bias was assessed using RoB2 and NOS tools. Quantitative data were pooled using fixed effect models, and qualitative data were pooled using narrative synthesis. Primary outcomes were asthma or asthma-like symptoms. The results were grouped according to population (general, asthmatic, and ibuprofen-hypersensitive), comparator type (active and non-active) and follow-up duration (short- and long-term).

**Results:**

Comparing ibuprofen with active comparators, there was no evidence of a higher risk associated with ibuprofen over both the short and long term in either the general or asthmatic population. Comparing ibuprofen use with no active alternative over a short-term follow-up, ibuprofen may provide protection against asthma-like symptoms in the general population when used to ease symptoms of fever or bronchiolitis. In contrast, it may cause asthma exacerbation for those with pre-existing asthma. However, in both populations, there were no clear long-term follow-up effects.

**Conclusions:**

Ibuprofen use in children had no elevated risk relative to active comparators. However, use in children with asthma may lead to asthma exacerbation. The results are driven by a very small number of influential studies, and research in several key clinical contexts is limited to single studies. Both clinical trials and observational studies are needed to understand the potential role of ibuprofen in childhood asthma pathogenesis.

**Supplementary Information:**

The online version contains supplementary material available at 10.1186/s12890-024-03179-3.

## Background

Asthma is a noncommunicable disease affecting approximately 235 million people worldwide and is characterised by inflammation and narrowing of the small airways in the lungs, leading to any combination of cough, wheeze, shortness of breath, and chest tightness [1]. The prevalence of asthma has increased in many countries in recent decades, especially among children, making asthma a serious global public health problem [2, 3]. The reason for increasing asthma prevalence in children is uncertain, but there is likely a complex interaction of multiple risk factors, including environmental (e.g., increased air pollution, changes to housing conditions) and lifestyle factors (e.g., decreased physical activity, changes in diet, increased childhood obesity) [4].

Increased early-life use of pharmacological agents, such as analgesics and antipyretics, could be causal factors in childhood asthma pathogenesis. Due to fears of a causal relationship between aspirin use and Reye’s syndrome [5] and the risk of aspirin-induced asthma [6], aspirin use in children has dramatically decreased in recent decades. Consequently, drugs such as ibuprofen and paracetamol have become increasingly popular for treating fever and pain in children. In the United Kingdom, the National Health Service describes both paracetamol and ibuprofen as safe for treating pain and high temperature in babies and children [7]. However, caution is advised for ibuprofen use in children with asthma [8], while no such warning is supplied for paracetamol [9], suggesting that ibuprofen may be linked to asthma development or exacerbation in those with pre-existing asthma.

Ibuprofen is a non-steroidal anti-inflammatory drug (NSAID) that is frequently prescribed or administered over-the-counter (OTC) to treat fever and pain. Links between childhood ibuprofen use and asthma development or exacerbation are being investigated [10–16]. Ibuprofen’s inhibition of the cyclooxygenase system can lead to activation of the lipoxygenase system, resulting in bronchospasm [6, 17], which could precipitate asthma. Additionally, empirical evidence exists demonstrating ibuprofen-induced asthma exacerbation in children with asthma and self-reported aspirin allergy [18].

Despite these points, two recent systematic reviews did not identify a risk difference between ibuprofen and paracetamol in asthma development or exacerbation in children [14, 16]. However, one of these reviews limited the scope to randomised controlled trials (RCTs) [14], and the other to a relatively narrow age range of less than 2 years [16], restricting the generalisability of the findings.

We conducted a systematic review to assess the association between ibuprofen administration in children and the risk of developing or exacerbating asthma. The aim was to expand on previous reviews by looking across the entire age range of childhood from 0 to 18 years, including both interventional and observational studies, and assessing the association separately for clinically distinct paediatric subpopulations: general, asthmatic, and ibuprofen-hypersensitive.

## Methods

### Protocol development

We registered our review on PROSPERO on 8 July 2022 (CRD42022344838). The protocol was written according to PRISMA-P guidelines [19, 20] and made publicly available on OSF prior to registration with PROSPERO. Further methodological details can be found in our online protocol (10.17605/OSF.IO/Z37KW).

### Eligibility criteria

A full list of eligibility criteria is provided in Supplementary Methods S1.1 (Supplementary Tables 1–2). The numeric results from studies included in our review were grouped by population for synthesis: (i) general population of children (i.e., studies not limiting eligibility to specific clinical subpopulations; however, some study-specific exclusion will always occur, for example, children with severe asthma, ibuprofen hypersensitivity, or other contraindications for safety reasons; children with conditions that could interfere with ibuprofen administration or absorption, such as inability to swallow or frequent vomiting; children receiving treatments that could interfere with the outcome assessment, such as leukotriene receptor antagonist and other anti-asthmatic treatments); (ii) children with asthma; and (iii) children with ibuprofen hypersensitivity.

### Search strategy

We searched six bibliographic databases (MEDLINE, Embase, Cochrane Library, CINAHL, Web Of Science, Scopus) to identify records on 21-May-2022, and our searches were independently peer-reviewed using the PRESS Checklist [21, 22] by an outreach librarian at the Bodleian Health Care Libraries, University of Oxford (10.17605/OSF.IO/R3AV6). All search strategies are provided in full in Supplementary Methods S1.2. Additional information sources included relevant reviews that were identified during screening [10–16] and backwards citation searching using the citationchaser tool [23]. EPPI-Reviewer [24] was used for de-duplication, and screening was performed independently in duplicate, with disagreements settled by discussion between both reviewers.

### Data extraction and bias assessment

Data extraction and bias assessment were performed by one reviewer and then verified by a second reviewer, with disagreements settled by discussion. Our primary outcomes of interest were asthma, asthma-like symptoms, or asthma exacerbation [2]. For risk of bias assessment, the Cochrane risk of bias tool (RoB2) was used for RCTs [25], and the Newcastle-Ottawa Scale (NOS) [26] was used for observational studies. The results from these assessments were used to decide which studies to include in primary syntheses (Supplementary Figs. 1–2). Our approach to assessing meta-biases (outcome reporting and publication biases) is detailed in Supplementary Methods S1.3.

### Data synthesis

A narrative synthesis was performed when outcomes were too heterogeneous to synthesise quantitatively. Otherwise, meta-analysis was performed using the R package *meta* [27]. Given the sparsity of the data for quantitative synthesis, we report the common effect model results as primary results. For completion, we report additional analysis outputs, e.g., both odds and risk ratios; both common and random effects model effect sizes; I^2^, tau^2^, and chi^2^ for heterogeneity. Due to the sparsity of the results, subgroup analyses were not performed.

For meta-analysis of dichotomous data, ORs were pooled using Peto’s method [28] due to zero events in some arms. Where multiple outcomes from a study were available, the primary analysis was performed by selecting the outcomes with the expected lowest risk of bias. To test the robustness of the primary analysis, sensitivity analyses were performed using alternative combinations of studies’ numeric results.

## Results

### Study selection characteristics

**Fig. 1:**
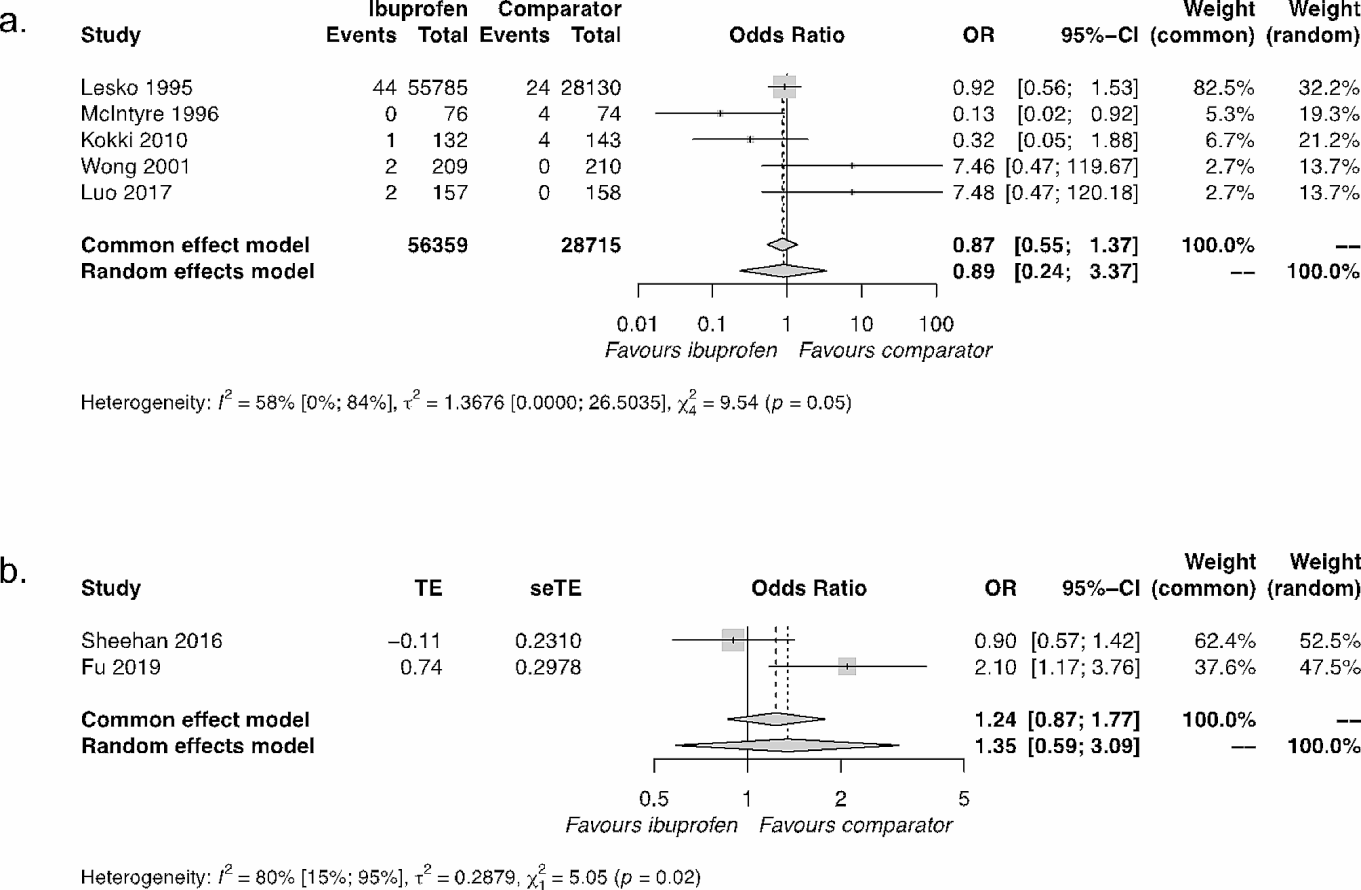
Synthesis of results of ibuprofen versus active comparators. The active comparator for Kokki 2010 was ketoprofen; for all other studies, the active comparator was paracetamol. (**a**) General population of children over a short duration. (**b**) Children with asthma over a long duration. Abbreviations: OR = odds ratio; 95% CI = 95% confidence interval

Of the 820 records screened, 18 relevant studies were identified, with a further 6 from relevant reviews (Supplementary Fig. 3). The study characteristics for all 24 studies are summarised in Table 1. Relevant numeric results were grouped by population: (i) general population of children (Table 2), (ii) children with asthma (Table 3), and (iii) children with ibuprofen hypersensitivity (Table 4). For the general population and children with asthma, data synthesis was performed for (i) ibuprofen versus an active comparator (Fig. 1) and (ii) ibuprofen versus baseline (i.e., children not taking an alternative antipyretic or analgesic). To increase homogeneity, the results were also grouped based on the duration of follow-up, in line with a recent similar systematic review [16]: short duration of ≤ 28 days or long duration of > 28 days.

**Table 1:** Study characteristics

Author (year)	Country	Study design	Comparator	Duration	Population health status	Reason for ibuprofen use	Age
Lesko (1995)	USA	Int, RCT, IRPG	Paracetamol	28 days	General	Fever	6 months – 12 years
Lesko (1999)	USA	Int, RCT, IRPG	Paracetamol	28 days	General	Fever	6 months – 2 years
McIntyre (1996)	UK	Int, RCT, IRPG	Paracetamol	3 days	General	Fever	2 months – 12 years
Kokki (2010)	France UK	Int, RCT, IRPG	Ketoprofen	4 days	General	Fever	6 months – 6 years
Wong (2001)	Brazil Argentina Chile Mexico	Int, RCT, IRPG	Paracetamol	14 days	General	Fever	6 months – 6 years
Luo (2017)	China	Int, RCT, IRPG	Paracetamol	5 days	General	Fever	6 months – 5 years
Walsh (2018)	USA	Obs, Cohort	Paracetamol Baseline	14 days 1 year	General	Bronchiolitis	0–12 months
Matok (2017)	Israel	Obs, Cross-sectional	Baseline	length of current febrile illness (unspecified)	General	Fever	6 months – 6 years
Sordillo (2015)	USA	Obs, Cohort	Baseline	3–5 years 7–10 years	General	Not specified	Infants: 0–1 year; children 3–5 years and 7–10 years
Lesko (2002)	USA	Int, RCT, IRPG	Paracetamol	28 days	Asthmatic	Fever	6 months – 12 years
Sheehan (2016)	USA	Int, RCT, IRPG	Paracetamol	46 weeks	Asthmatic (mild persistent asthma)	Fever or pain	12 months – 59 months
Fu (2019)	Taiwan	Obs, Cohort	Paracetamol	52 weeks	Asthmatic (persistent asthma)	Not specified	1–5 years
Lo (2016)	Taiwan	Obs, Cohort	Baseline	1–2 days > 12 weeks	Asthmatic	Fever or pain	0–18 years
Corzo (2014)	Spain	Int, DPT	n/a	< 1 day	Ibuprofen hypersensitive	Hypersensitivity diagnosis	1–14 years
Guvenir (2015)	Turkey	Int, DPT	n/a	< 1 day	Ibuprofen hypersensitive	Hypersensitivity diagnosis	11 months – 16 years
Ertoy Karagol (2015)	Turkey	Int, DPT	n/a	< 1 day	Ibuprofen hypersensitive	Hypersensitivity diagnosis	13–17 years
Yilmaz Topal (2020)	Turkey	Int, DPT	n/a	< 1 day	Ibuprofen hypersensitive	Hypersensitivity diagnosis	4–11 years (IQR)
Debley (2005)	USA	Int, RCT, Crossover	Placebo	< 1 day (2 to 7 days between visits)	Asthmatic (mild or moderate persistent asthma)	Bronchoprovocation testing	6–18 years
Su (2015)	Taiwan	Int, non-RCT	Healthy controls Allergic controls	3 days	Asthmatic (mild to moderate stable asthma with self-reported aspirin allergy)	Ibuprofen sensitivity / pulmonary function testing	9–10 years
Menendez (1998)	USA	Obs, Case report	n/a	n/a	Moderately severe asthma	Headache	14 years
Goraya (2001)	India	Obs, Case report	n/a	n/a	Potential mild intermittent asthma	Fever due to mild upper respiratory illness	2 years
Palmer (2005)	Australia	Obs, Case report	n/a	n/a	Severe asthma, allergic rhinitis	Postoperative pain management	17 years
King (2016)	Ireland	Obs, Case report	n/a	n/a	Atopic asthma, allergic rhinitis	Post-dental extraction analgesia, followed by ibuprofen challenge for hypersensitivity testing	13 years
Malmstrom (2007)	Finland	Obs, Case series	n/a	n/a	Severe asthma, known allergy to ibuprofen	Not provided	Not provided (between 12-19.5 years)

Abbreviations: Int = interventional; Obs = observational; RCT = randomised controlled trial; IRPG = individually-randomised parallel groups; DPT = drug provocation test; non-RCT = non-randomised controlled trial; USA = United States of America; UK = United Kingdom

**Table 2:** Results for the general population of children. For all results, values less than 1 indicate ibuprofen to be favourable, and values greater than 1 indicate the comparator to be favourable

Author (year)	Comparator	Design	Dose	Duration	Outcome	Sample size	Result
Lesko (1995)	Paracetamol	Int, RCT	Ibuprofen: 5–10 mg/kg Paracetamol: 12 mg/kg	Short (28 days)	Hospitalisation with asthma discharge diagnosis	83,915	Ibuprofen > Comparator RR = 0.92 [0.56, 1.52] OR = 0.92 [0.56, 1.53]
Lesko (1999)	Paracetamol	Int, RCT	Ibuprofen: 5–10 mg/kg Paracetamol: 12 mg/kg	Short (28 days)	Hospitalisation with asthma/bronchiolitis discharge diagnosis	27,065	Ibuprofen > Comparator RR = 0.87 [0.53, 1.44] OR = 0.87 [0.52, 1.45]
McIntyre (1996)	Paracetamol	Int, RCT	Ibuprofen: 20 mg/kg Paracetamol: 50 mg/kg	Short (3 days)	Respiratory distress, cough, or asthma	150	Ibuprofen > Comparator RR = 0.19 [0.01, 3.99] OR = 0.13 [0.01, 2.10]
Kokki (2010)	Ketoprofen	Int, RCT	Ibuprofen: 5 mg/kg Ketoprofen: 0.5 mg/kg	Short (4 days)	Cough	275	Ibuprofen > Comparator RR = 0.27 [0.03, 2.39] OR = 0.32 [0.05, 1.88]
Wong (2001)	Paracetamol	Int, RCT	Ibuprofen: 5–10 mg/kg Paracetamol: 12 mg/kg	Short (14 days)	Wheezing or bronchospasm	419	Ibuprofen < Comparator RR = 5.02 [0.24, 104.01] OR = 7.46 [0.47, 119.67]
Luo (2017)	Paracetamol	Int, RCT	Ibuprofen: 10 mg/kg Paracetamol: 10 mg/kg	Short (5 days)	Asthma	315	Ibuprofen < Comparator RR = 5.03 [0.24, 103.97] OR = 7.48 [0.47, 120.18]
Walsh (2018)	Paracetamol	Obs, Cohort	Not provided	Short (14 days)	HCP visit for wheezing illness consistent with bronchiolitis or asthma	13,637	Ibuprofen > Comparator aIRR = 0.98 [0.77, 1.26]
Walsh (2018)	Paracetamol	Obs, Cohort	Not provided	Long (1 year)	HCP visit for wheezing illness consistent with bronchiolitis or asthma	10,198	Ibuprofen > Comparator aIRR = 0.82 [0.70, 0.95] *
Matok (2017)	Baseline	Obs, Cross-sectional	Not provided	Short (length of current febrile illness, unspecified)	Wheezing	347	Ibuprofen > Comparator aOR = 0.36 [0.13, 0.96] *
Walsh (2018)	Baseline	Obs, Cohort	Not provided	Short (14 days)	HCP visit for wheezing illness consistent with bronchiolitis or asthma	15,787	Ibuprofen > Comparator aIRR = 0.15 [0.14, 0.16] *
Walsh (2018)	Baseline	Obs, Cohort	Not provided	Long (1 year)	HCP visit for wheezing illness consistent with bronchiolitis or asthma	11,317	Ibuprofen > Comparator aIRR = 0.18 [0.12 0.27] *
Sordillo (2015)	Baseline	Obs, Cohort	Not provided	Long (3–5 years)	Asthma	1,419	Ibuprofen < Comparator aOR = 1.20 [1.02, 1.40] *
Sordillo (2015)	Baseline	Obs, Cohort	Not provided	Long (7–10 years)	Asthma	1,220	Ibuprofen = Comparator aOR = 1.00 [0.83, 1.21]

Abbreviations: Int = interventional; Obs = observational; RCT = randomised controlled trial; RR = risk ratio; OR = odds ratio; aOR = adjusted odds ratio; IRR = incident rate ratio; aIRR = adjusted incident rate ratio; * = statistically significant

**Table 3:** Results for children with asthma. For all results, values less than 1 indicate ibuprofen to be favourable, and values greater than 1 indicate the comparator to be favourable

Author (year)	Comparator	Design	Dose	Duration	Outcome	Sample size	Result
Lesko (2002)	Paracetamol	Int, RCT	Ibuprofen: 5–10 mg/kg Paracetamol: 12 mg/kg	Short (28 days)	Hospitalisation with asthma	1,879	Ibuprofen > Comparator RR = 0.63 [0.25, 1.6]
Sheehan (2016)	Paracetamol	Int, RCT	Ibuprofen: 9.4 mg/kg Paracetamol: 15 mg/kg	Long (46 weeks)	Asthma exacerbation	300	Ibuprofen > Comparator RR = 0.95 [0.75, 1.20] OR = 0.90 [ 0.57, 1.41]
Fu (2019)	Paracetamol	Obs, Cohort	Not provided	Long (52 weeks)	Asthma exacerbation	983	Ibuprofen < Comparator aOR = 2.10 [1.17, 3.76] *
Lo (2016)	Baseline	Obs, Cohort	Not provided	Short (1–2 days)	Asthma exacerbation	52	Ibuprofen < Comparator aOR = 3.65 [1.98, 6.74] *
Lo (2016)	Baseline	Obs, Cohort	Not provided	Long (> 12 weeks)	Asthma exacerbation	93	Ibuprofen > Comparator aOR = 0.90 [0.62, 1.30]

Abbreviations: Int = interventional; Obs = observational; RCT = randomised controlled trial; RR = risk ratio; OR = odds ratio; aOR = adjusted odds ratio; IRR = incident rate ratio; aIRR = adjusted incident rate ratio; * = statistically significant

**Table 4:** Results for children with ibuprofen hypersensitivity

Author (year)	Design	Duration	Outcome	Resp. AEs	Sample size	Result
Corzo (2014)	Int, DPT	< 1 day	Asthma	1	41	2.44%
Guvenir (2015)	Int, DPT	< 1 day	Cough, dyspnoea	1	9	11.11%
Ertoy Karagol (2015)	Int, DPT	< 1 day	Dyspnoea, coughing, wheezing	2	3	66.67%
Yilmaz Topal (2020)	Int, DPT	< 1 day	Respiratory distress	6	27	22.22%
				10	80	12.5%

Abbreviations: Int = interventional; DPT = drug provocation trial

### General population

In total, 13 numeric results from 9 studies relevant to assessing ibuprofen use in a general population of children were identified [29–37] (Table 2).

#### Ibuprofen versus active comparator

There were six results from six interventional studies (all RCTs) and two results from one observational cohort study that compared ibuprofen use with an active comparator in the general population. The main active comparator was paracetamol, with one study [29] using ketoprofen (Table 2). The durations of study for the interventional RCT were all short (≤ 28 days). Two of these results were from publications based on the same dataset, the Boston University Fever Study [30, 31], of which the original publication was selected for primary analysis.

The synthesis of five results comparing ibuprofen with active comparators (four paracetamol, one ketoprofen) resulted in a common effect OR = 0.87; 95% CI=[0.55, 1.37], demonstrating a lack of significant difference between ibuprofen and active comparators (Fig. 1a). Our sensitivity analyses were in agreement with this primary result (Supplementary Fig. 4).

A single observational study [36] assessed ibuprofen relative to paracetamol over both short and long durations (Table 2) in a general population of children. Over a short duration (14 days), no significant difference in wheezing was identified, but over a long duration (1 year), they observed a significant advantage to ibuprofen over paracetamol, with a reduction in health care practitioner visits for wheezing illness consistent with bronchiolitis or asthma.

Taken together, these interventional and observational results suggest that there is no difference between ibuprofen and active comparators in the general population over a short duration (≤ 28 days). This finding is driven largely by a single study, the Boston University Fever Study [31], conducted almost 30 years ago on a large sample (*n* = 83,915) of children aged 6 months to 12 years. Over longer follow-up durations of one year, there is evidence from only a single cohort study [36] to suggest that there may be a reduction in wheezing when ibuprofen is prescribed, rather than paracetamol, for a first episode of bronchiolitis in children aged 0–12 months.

#### Ibuprofen versus baseline

Five numeric results from three studies relevant to assessing ibuprofen relative to baseline (children not taking an alternative antipyretic or analgesic) in the general population were identified (Table 2). All outcomes were from observational studies. Due to the sparsity and substantive heterogeneity of the results, quantitative synthesis was not possible.

Two studies looked at general populations over short durations (≤ 28 days) [33, 36]. Both studies suggest that ibuprofen might decrease wheezing when taken for either acute febrile illness or bronchiolitis (Table 2).

Two studies looked at general populations of children over long durations [35, 36] and produced conflicting results. One study [36] compared those prescribed ibuprofen for a first episode of bronchiolitis to those not prescribed ibuprofen (or another drug) and followed up participants over a 1-year duration, observing a positive impact of ibuprofen prescription. The second study [35] compared children administered ibuprofen to those not administered ibuprofen during the first postnatal year and followed-up participants at a 3–5 year duration, observing a negative impact of ibuprofen on asthma development, and at a 7–10 year duration, observing no difference between cohorts (Table 2).

Taken together, ibuprofen use in the general population of children during acute febrile illness or bronchiolitis might decrease wheezing when assessed in the short-term (≤ 28 days), with both observational studies reporting strong significant effects (Table 2). Over longer durations, the two observational studies identified in this review have substantive heterogeneity in design, analysis, and outcome, preventing meaningful synthesis. Additionally, their numeric findings are inconsistent (Table 2).

### Asthmatic population

Five numeric results from four studies relevant to assessing ibuprofen in asthmatic paediatric populations were identified [38–41] (Table 3).

#### Ibuprofen versus active comparator

Three results across three studies compared ibuprofen with an active comparator (paracetamol in all cases) in asthmatic populations (Table 3). One interventional study assessed outcomes over a short duration [39] and found no difference between treatments. While further analyses in this paper did suggest a favourable outcome for ibuprofen relative to paracetamol, the results from this second post-hoc Boston Fever Study report are at very high risk of bias in the selection of the reported result (Supplementary Fig. 2).

Two studies looked at the comparison between ibuprofen and paracetamol in asthmatic populations over long durations [38, 41]. The RCT study [41] identified no difference between drugs (OR = 0.90 [ 0.57, 1.41]). In contrast, the observational cohort study [38] identified a significant disadvantage for ibuprofen relative to paracetamol in asthmatic populations (aOR = 2.10 [1.17, 3.76]). These conflicting results for ibuprofen relative to paracetamol in asthmatic populations over long durations are challenging to resolve due to the different experimental designs. However, there are also several similarities in their designs: use of the same active comparator, inclusion of asthmatic populations of children with similar age ranges (Sheehan: 1–4.9 years; Fu: 1–5 years) over similar follow-up durations (Sheehan: 46 weeks; Fu: 52 weeks), and use of asthma exacerbation as the outcome. As an exploratory analysis, we synthesised these results, which resulted in a common effect OR = 1.24; 95% CI=[0.87, 1.77], suggesting an overall non-significant effect, which is consistent with the RCT study result alone (Fig. 1b).

Taken together, these interventional and observational results suggest that there is no difference in asthma exacerbation between ibuprofen and paracetamol in asthmatic populations over short or long durations.

#### Ibuprofen versus baseline

Only a single study looked at an asthmatic population over both short and long durations [40]. Over a short duration, this study found that ibuprofen increased asthma exacerbation. Over a long duration, they found no effect of ibuprofen on asthma exacerbation in the asthmatic population.

### Ibuprofen hypersensitive population

Four drug provocation studies were identified that studied ibuprofen-hypersensitive children where ibuprofen was ingested and adverse events reported as part of hypersensitivity diagnosis [42–45]. A range of respiratory adverse effects were reported that included asthma, coughing, wheezing, dyspnoea, and respiratory distress (Table 4). Across the four studies, there was a total of 10 children with respiratory adverse events reported in a total of 80 children. Thus, in children with ibuprofen hypersensitivity, the average rate of respiratory adverse events following ibuprofen ingestion was 12.5%.

### Unsynthesised papers

Seven studies were identified that reported the relationship between ibuprofen and asthma in children, which were not synthesised in this review [18, 46–51]: five studies reported on single cases, and two group analysis studies had substantive differences in methodology and outcomes relative to other studies included in this review.

One crossover RCT [46] assessed the prevalence of ibuprofen-sensitive asthma in children with mild or moderate persistent asthma using bronchoprovocation challenge and found a prevalence of 2%. Another non-randomised controlled study [18] assessed the impact of short-term ibuprofen treatment on pulmonary function in children with mild to moderate stable asthma and self-reported aspirin allergy. Relative to a healthy control group, the asthmatic group exhibited a drop in FEV1 (forced expiratory volume in the first second) of 18.85% and an increase in FeNO (fractional exhaled nitric oxide) of 20.76 ppb. A summary of the results from these two studies is provided in Supplementary Table 3.

Four case reports of severe adverse events to ibuprofen were identified [47, 48, 50, 51], and in all cases, the children had pre-existing asthma. Last, in a case series of fatal asthma in Finland, a single death due to ibuprofen ingestion was reported in a child with severe asthma and a known allergy to ibuprofen [49].

## Discussion

Here, we assessed the association between ibuprofen use and asthma in children aged 0–18 years. Both observational and interventional studies were reviewed in the general population as well as the asthmatic population. Studies that benchmarked ibuprofen against an active comparator almost exclusively used paracetamol, and in both populations of children, the combined evidence suggested no difference in asthma-related adverse events between ibuprofen and paracetamol (or ketoprofen) use. A single observational study suggested a potential benefit of ibuprofen over paracetamol prescription in response to bronchiolitis in the general paediatric population after a one-year follow-up. When ibuprofen use was assessed relative to no alternative drug administration, differences emerged between the general and asthmatic populations. In the short-term follow-up (1–14 days) to ibuprofen use, two observational studies reported favourable effects in the general population, while one observational and one interventional study observed unfavourable effects in the asthmatic population. Over a longer follow-up period (12 weeks to 10 years), no clear effect emerged for either population.

The majority of research on the association between ibuprofen use and asthma-related adverse events in children has been conducted in the general population, benchmarked relative to paracetamol, and participants followed-up over a short duration [29–32, 34, 36, 37]. The aggregate result from five RCTs conducted in this context is driven primarily by the Boston University Fever Study [31], conducted almost 30 years ago on children aged 6 months to 12 years. While a single observational study [36] conducted five years ago corroborates this finding, research is sparse. Furthermore, only a single study comparing ibuprofen with paracetamol use with a short-term follow-up was conducted in children with asthma [39], and this study was a second post-hoc analysis publication of the same Boston University Fever Study dataset. Given the increased vulnerability of the asthmatic population to respiratory adverse events from ibuprofen use that was observed in our review, there is a clear lack of research comparing the short-term effects of ibuprofen relative to alternative analgesics and antipyretics such as paracetamol in children with asthma.

Two studies [38, 41] assessing differences between ibuprofen and paracetamol use over longer follow-up periods in asthmatic populations report conflicting results. Due to several study similarities, we tentatively synthesised the two results, and no aggregate difference between ibuprofen and paracetamol was observed. However, in the RCT [41], the median dose of trial medication (ibuprofen or paracetamol) was 5.5 doses (IQR = 1–15) and matched between trial arms. In the retrospective cohort study [38], it could not be determined by the original investigators whether patients took the medication prescribed. Additionally, the observational study did not control for upper respiratory tract infections, a well-documented source of confounding by indication [35, 52], which were not well-matched between the ibuprofen and paracetamol cohorts. For these reasons, the RCT finding alone or the synthesised outcome of no difference between drugs seems most justifiable.

Comparing the asthmatic and general populations for short-term asthma-relevant outcomes after ibuprofen use, no conflicts in results were observed. The two observational studies in the general population [33, 36] both observed reductions in asthma-related outcomes, while one observational [40] and one interventional [18] study in the asthmatic population both observed increases in asthma-related outcomes. These findings highlight the importance of avoiding naïve pooling of results from studies in these different paediatric populations.

It is noteworthy that all RCTs reviewed compared ibuprofen with an active comparator. Of the studies comparing ibuprofen with a baseline of no alternative drug, three were cohort studies [35, 36, 40], and one was cross-sectional [33]. One non-randomised interventional study [18] compared an asthmatic sample with a healthy control sample. This highlights one of the limitations of the RCT design approach in assessing adverse events in the youngest children [53, 54]. As a recent RCT feasibility study found [55], almost three quarters of parents surveyed described the use of a placebo comparator treatment as unacceptable for treating their child’s fever or pain. This ethical unacceptability of using a placebo arm in clinical trials for treating pain and fever in young children [55, 56] introduces an ambiguity into these active comparator RCT studies, as a lack of difference among active comparators does not exclude the possibility that both ibuprofen and active comparator use may be associated with parallel increases in asthma exacerbations [41, 56]. It has been argued that, given that ibuprofen and paracetamol have different mechanisms of action, it is unlikely that their use could be associated with similar increases in the rate of asthma-related complications that are known to be determined by disparate mechanisms of disease [41, 56]. However, this speculation requires careful examination and empirical support. Observational studies with comparator groups in which an active treatment was not prescribed or taken can be used as a baseline control to assess the impact of ibuprofen alone, acknowledging the challenges of inferring causality in observational studies. It is these advantages and disadvantages of both RCTs and observational designs that require a review of the association between ibuprofen use and asthma-related outcomes in children to consider and attempt to synthesise all study design types. This feature of our review adds substantially to two recent systematic reviews in this area [14, 56] that either limited the study designs to RCTs [14] or limited the population to those under 2 years [56].

We identified four drug provocation trials in which ibuprofen hypersensitivity was confirmed in children by controlled administration of ibuprofen [42–45] and respiratory adverse events were recorded. The average percentage of children with confirmed ibuprofen hypersensitivity who displayed respiratory adverse events was 12.5%. Relative to other adverse events, such as angio-oedema and urticaria (which were by far the most common adverse events), asthma and asthma-like respiratory events were less commonly reported. While adverse respiratory reactions to ibuprofen ingestion in those with ibuprofen hypersensitivity can be quite severe, as reported in a handful of case reports [47, 48, 50, 51], fatalities appear to be very rare. In this review, only a single case of ibuprofen-induced asthma fatality was identified [49].

The number of studies in this review that were relevant to important clinical populations and contexts was unfortunately sparse. Only a single publication was identified for each of the following three contexts: the general population where ibuprofen is compared with an active comparator with a follow-up duration longer than 1 month [36]; the asthmatic population where ibuprofen is compared with an active comparator with a short-term follow-up [39]; and the asthmatic population where ibuprofen is compared with a baseline of no active comparator with a follow-up duration longer than 1 month [40]. These limitations hinder the generalisability of findings to several important clinical contexts and are an ongoing issue to be addressed.

## Conclusion

Here, we found that research is most lacking for populations of children with pre-existing asthma, who are the population at most risk for potential respiratory adverse events following ibuprofen use. Our review highlights the importance of assessing both interventional and observational studies and analysing the general population and asthmatic population separately. Continued investigation into the role of early-life ibuprofen use and its short-term and long-term impact on childhood asthma is needed.

### Electronic supplementary material

Below is the link to the electronic supplementary material.

Supplementary Material 1

## Data Availability

All data (data collection form, risk of bias assessment forms, and data used for all analyses) are publicly available on the project’s OSF site: 10.17605/OSF.IO/ZBDS7. All code used for the meta-analysis is publicly available on Zenodo: 10.5281/zenodo.11258287.
