# Supplementary material for: The association between ibuprofen administration in children and the risk of developing or exacerbating asthma: a systematic review and meta-analysis
Source: BMC Pulm Med. 2024 Aug 26;24:412. doi: 10.1186/s12890-024-03179-3 (PMC11348613; doi:10.1186/s12890-024-03179-3)
Supplement: Supplementary file 1 — Supplementary Material 1 [file 12890_2024_3179_MOESM1_ESM.docx]

**Supplementary Information**

**The association between ibuprofen administration in children and the risk of developing or exacerbating asthma: a systematic review and meta-analysis**

**Baxter et al.**

**S1 Supplementary Methods**

**S1.1 Eligibility criteria**

***Supplementary Table 1: Inclusion criteria***

| **Population** | Children aged 0-18 years |
| --- | --- |
| **Intervention/Exposure** | Any OTC (over-the-counter) or non-OTC doses of oral or any other form of ibuprofen (suppositories, etc) for short or long duration of time for fever or any other indication including pain in children. |
| **Comparator** | Placebo or any doses of paracetamol or other NSAIDs. |
| **Outcome** | 1. Worsening of asthma or asthma-like symptoms in children previously identified as asthmatic.  2. Development of asthma or asthma-like symptoms or diagnosis of asthma in previously non-asthmatic patients. |
| **Study design** | Any study design of primary empirical study (controlled, uncontrolled, interventional, observational). |
| **Report characteristics** | Primary empirical research (e.g. randomised and non-randomised controlled trials, cohort studies, case-control studies, cross-sectional studies, case series, case reports); full peer-reviewed publications; any year of dissemination; any publication language. |

***Supplementary Table 2: Exclusion criteria***

| **Population** | Prenatal humans, humans aged over 18 years, non-humans. |
| --- | --- |
| **Intervention/Exposure** | n/a |
| **Comparator** | Narcotics/opioids. |
| **Outcome** | n/a adverse events related to ibuprofen that are non-respiratory (e.g. gastrointestinal, cardiovascular). |
| **Study** | n/a |
| **Report characteristics** | Secondary literature (e.g. reviews, book chapters); non-empirical research (e.g. commentaries, opinions, perspectives); unpublished grey literature (e.g. conference abstracts, posters). |

**S1.2 Search strategies**

The six bibliographic databases that we searched were: CINAHL (EBSCO Industries), Cochrane Library (John Wiley & Sons), Embase (Ovid Technologies, Inc), MEDLINE (Ovid Technologies, Inc), Scopus (Elsevier), and Web of Science (Clarivate Analytics). The search was initially developed in MEDLINE, and was composed of three blocks: (i) population of interest (children 0-18 years, excluding prenatal humans), (ii) intervention or exposure of interest (ibuprofen), and (iii) outcome of interest (asthma or asthma-like symptoms). No filters, limits or further restrictions were used. The search was then translated to other databases using the Systematic Review Accelerator Polyglot tool (1).

*MEDLINE (Ovid Technologies, Inc):*

(exp "infant, newborn"/ or exp infant/ or exp child/ or exp "child, preschool"/ or exp adolescent/ or newborn*.ti,ab. or neonat*.ti,ab. or infant*.ti,ab. or baby*.ti,ab. or babies*.ti,ab. or toddler*.ti,ab. or child*.ti,ab. or preschool*.ti,ab. or adolescen*.ti,ab. or teenage*.ti,ab.) AND (exp ibuprofen/ or ibuprofen*.ti,ab.) AND (exp asthma/ or exp "bronchial spasm"/ or exp bronchoconstriction/ or exp bronchiolitis/ or exp dyspnea/ or asthma*.ti,ab. or "bronchial spasm*".ti,ab. or bronchospasm*.ti,ab. or bronchoconstriction*.ti,ab. or bronchiolitis*.ti,ab. or dyspnea*.ti,ab. or dyspnoea*.ti,ab. Or wheez*.ti,ab.)

*Embase (Ovid Technologies, Inc):*

(exp newborn/ or exp infant/ or exp child/ or exp "preschool child"/ or exp adolescent/ or newborn*.ti,ab. or neonat*.ti,ab. or infant*.ti,ab. or baby*.ti,ab. or babies*.ti,ab. or toddler*.ti,ab. or child*.ti,ab. or preschool*.ti,ab. or adolescen*.ti,ab. or teenage*.ti,ab.) AND (exp ibuprofen/ or ibuprofen*.ti,ab.) AND (exp asthma/ or exp bronchospasm/ or exp bronchoconstriction/ or exp bronchiolitis/ or exp dyspnea/ or asthma*.ti,ab. or "bronchial spasm*".ti,ab. or bronchospasm*.ti,ab. or bronchoconstriction*.ti,ab. or bronchiolitis*.ti,ab. or dyspnea*.ti,ab. or dyspnoea*.ti,ab. or wheez*.ti,ab.)

*Cochrane Library (John Wiley & Sons):*

([mh "infant, newborn"] OR [mh infant] OR [mh child] OR [mh "child, preschool"] OR [mh adolescent] OR newborn*:ti,ab OR neonat*:ti,ab OR infant*:ti,ab OR baby*:ti,ab OR babies*:ti,ab OR toddler*:ti,ab OR child*:ti,ab OR preschool*:ti,ab OR adolescen*:ti,ab OR teenage*:ti,ab) AND ([mh ibuprofen] OR ibuprofen*:ti,ab) AND ([mh asthma] OR [mh "bronchial spasm"] OR [mh bronchoconstriction] OR [mh bronchiolitis] OR [mh dyspnea] OR asthma*:ti,ab OR ("bronchial" NEXT spasm*):ti,ab OR bronchospasm*:ti,ab OR bronchoconstriction*:ti,ab OR bronchiolitis*:ti,ab OR dyspnea*:ti,ab OR dyspnoea*:ti,ab OR wheez*:ti,ab)

*CINAHL (EBSCO Industries):*

((MH "infant, newborn+") OR (MH infant+) OR (MH child+) OR (MH "child, preschool+") OR (MH adolescence+) OR (TI newborn* OR AB newborn*) OR (TI neonat* OR AB neonat*) OR (TI infant* OR AB infant*) OR (TI baby* OR AB baby*) OR (TI babies* OR AB babies*) OR (TI toddler* OR AB toddler*) OR (TI child* OR AB child*) OR (TI preschool* OR AB preschool*) OR (TI adolescen* OR AB adolescen*) OR (TI teenage* OR AB teenage*)) AND ((MH ibuprofen+) OR (TI ibuprofen* OR AB ibuprofen*)) AND ((MH asthma+) OR (MH "bronchial spasm+") OR (MH bronchoconstriction+) OR (MH bronchiolitis+) OR (MH dyspnea+) OR (TI asthma* OR AB asthma*) OR (TI "bronchial spasm*" OR AB "bronchial spasm*") OR (TI bronchospasm* OR AB bronchospasm*) OR (TI bronchoconstriction* OR AB bronchoconstriction*) OR (TI bronchiolitis* OR AB bronchiolitis*) OR (TI dyspnea* OR AB dyspnea*) OR (TI dyspnoea* OR AB dyspnoea*) OR (TI wheez* OR AB wheez*))

*Web Of Science Core Collection (Clarivate Analytics):*

((TI=newborn* OR AB=newborn*) OR (TI=neonat* OR AB=neonat*) OR (TI=infant* OR AB=infant*) OR (TI=baby* OR AB=baby*) OR (TI=babies* OR AB=babies*) OR (TI=toddler* OR AB=toddler*) OR (TI=child* OR AB=child*) OR (TI=preschool* OR AB=preschool*) OR (TI=adolescen* OR AB=adolescen*) OR (TI=teenage* OR AB=teenage*)) AND (TI=ibuprofen* OR AB=ibuprofen*) AND ((TI=asthma* OR AB=asthma*) OR (TI="bronchial spasm*" OR AB="bronchial spasm*") OR (TI=bronchospasm* OR AB=bronchospasm*) OR (TI=bronchoconstriction* OR AB=bronchoconstriction*) OR (TI=bronchiolitis* OR AB=bronchiolitis*) OR (TI=dyspnea* OR AB=dyspnea*) OR (TI=dyspnoea* OR AB=dyspnoea*) OR (TI=wheez* OR AB=wheez*))

*Scopus (Elsevier):*

(TITLE-ABS(newborn*) OR TITLE-ABS(neonat*) OR TITLE-ABS(infant*) OR TITLE-ABS(baby*) OR TITLE-ABS(babies*) OR TITLE-ABS(toddler*) OR TITLE-ABS(child*) OR TITLE-ABS(preschool*) OR TITLE-ABS(adolescen*) OR TITLE-ABS(teenage*)) AND (TITLE-ABS(ibuprofen*)) AND (TITLE-ABS(asthma*) OR TITLE-ABS("bronchial spasm*") OR TITLE-ABS(bronchospasm*) OR TITLE-ABS(bronchoconstriction*) OR TITLE-ABS(bronchiolitis*) OR TITLE-ABS(dyspnea*) OR TITLE-ABS(dyspnoea*) OR TITLE-ABS(wheez*))

**S1.3 Meta-bias assessment**

Prisma guidelines specify two meta-biases to address in systematic reviews: outcome reporting bias and publication bias. Outcome reporting bias forms Domain 5 of the RoB2 tool and therefore will be reported as part of the RoB2 result for RCTs. Outcome reporting bias is not assessed as part of NOS. Adopting the RoB2 algorithm for assessing this bias, if the answer to the signalling question “Was the study analysed in accordance with a pre-specified plan?” is “No”, “Probably no”, or “No information provided”, then the minimum risk of bias would be “Some concerns”. Given the nature of observational studies, it is unlikely any study in this category will have a pre-specified analysis plan, and so there is likely to be at least some concerns of outcome reporting bias for all results here. This assumption was confirmed for all observational studies in this review, so no further outcome reporting bias was assessed, and this well-known limitation of observational studies is taken into account when interpreting the results.

Publication bias was estimated graphically by funnel plots as well as statistically using the Harbord test. Funnel plots and Harbord testing were performed using the R package *meta* (2). Publication bias testing requires numeric results to be of the same type and a minimum of approx. 10 observations are recommended for meaningful testing. Analysis of the general population, benchmarked relative to paracetamol, and participants followed-up over a short duration, had the greatest number of results (n=5). The non-significant results here (t = 0.11, df = 3, p-value = 0.9187) were not meaningful due to too low study number, and so, further publication bias assessments could not be meaningfully performed.

**S2 Supplementary Results**

***Supplementary Figure 1: Risk of bias assessment for RCTs.***

***Supplementary Figure 2: Risk of bias assessment for observational studies.*** *Abbreviations: aIRR = adjusted incident rate ratio, aOR = adjusted odds ratio.*

**Identification of studies via bibliographic databases, reviews, and citation searching**

Duplicate records removed: n = 313

Records identified from bibliographic databases: n = 1,133

CINAHL: n = 57

Cochrane: n = 38

Embase: n = 767

MEDLINE: n = 103

Scopus: n = 95

Web Of Science: n = 73

**Identification**

Reports sought for retrieval: n = 49

Reports not retrieved: n = 0

Records screened: n = 820

Records excluded: n = 771

**Screening**

Reports assessed for eligibility: n = 49

Reports excluded: n = 31

Association not assessed: n = 20

Wrong population: n = 5

Wrong outcome: n = 5

Duplicate case report: n = 1

**Included**

Reports identified from other sources:

Reviews: n = 6

Citation searching: n = 0

Reports of included studies: n = 24

Databases: n = 18

Reviews: n = 6

***Supplementary Figure 3: Study selection PRISMA flowchart.***

***Supplementary Figure 4: RCTs on a general population of children over a short duration (≤28 days) using an active comparator.*** *The active comparator for Kokki 2010 was ketoprofen; for all other studies, the active comparator was paracetamol. This is a sensitivity analysis for the analysis in Figure 1a.* ***a.*** *The original Boston University Fever Study (3) was replaced by the post-hoc study (4).* ***b-c.*** *Based on the risk of bias assessment (Figure S1), two studies at high risk of bias (5,6) were excluded, and the analyses with each Boston University Fever Study were recalculated.*

***Supplementary Table 3: Summary of unsynthesised group analysis studies.***

| **Author (year)** | **Comparator** | **Population** | **Design** | **Dose** | **Duration** | **Outcome** | **Sample size** | **Result** |
| --- | --- | --- | --- | --- | --- | --- | --- | --- |
| Debley (2005) | Placebo | Asthmatic | Int. | 10 mg/kg | Short  (<1 day) | Cough, dyspnoea | 100 | Prevalence = 2% [0.2%, 7%] |
| Su (2015) | Healthy controls  Allergic controls | Asthmatic | Int. | 2.5 mg/kg | Short  (3 days) | Asthma | 90 | Healthy controls:  FEV1 = –18.85%  FeNO = +20.76 ppb  Allergic controls:  FEV1 = –14.72%  FeNO = +4.73 ppb |

*Abbreviations: Int = interventional; FEV1 = forced expiratory volume in the first second; FeNO = fractional exhaled nitric oxide; ppb = parts per billion;*

**S3 Supplementary References**

1. Clark JM, Sanders S, Carter M, Honeyman D, Cleo G, Auld Y, et al. Improving the translation of search strategies using the Polyglot Search Translator: a randomized controlled trial. J Med Libr Assoc. 2020 Apr;108(2):195–207.

2. Balduzzi S, Rücker G, Schwarzer G. How to perform a meta-analysis with R: a practical tutorial. Evid Based Ment Health. 2019 Nov;22(4):153–60.

3. Lesko SM, Mitchell AA. An assessment of the safety of pediatric ibuprofen. A practitioner-based randomized clinical trial. JAMA. 1995 Mar 22;273(12):929–33.

4. Lesko SM, Mitchell AA. The safety of acetaminophen and ibuprofen among children younger than two years old. Pediatrics. 1999 Oct;104(4):e39.

5. Kokki H, Kokki M. Ketoprofen versus paracetamol (acetaminophen) or ibuprofen in the management of fever: results of two randomized, double-blind, double-dummy, parallel-group, repeated-dose, multicentre, phase III studies in children. Clin Drug Investig. 2010;30(6):375–86.

6. Luo S, Ran M, Luo Q, Shu M, Guo Q, Zhu Y, et al. Alternating Acetaminophen and Ibuprofen versus Monotherapies in Improvements of Distress and Reducing Refractory Fever in Febrile Children: A Randomized Controlled Trial. Paediatr Drugs. 2017 Oct;19(5):479–86.
